# Single-Incision Versus Conventional Laparoscopic Appendectomy: A Multi-Center Randomized Controlled Trial (SCAR trial)

**DOI:** 10.29337/ijsp.159

**Published:** 2021-08-30

**Authors:** Sung Il Kang, In Teak Woo, Sung Uk Bae, Chun-Seok Yang

**Affiliations:** 1 Department of Surgery, Yeungnam University Medical Center, Yeungnam University College of Medicine, Daegu, KR; 2 Department of Surgery, Pohang Medical Center, Pohang, KR; 3 Department of Surgery, Dongsan Medical Center, School of Medicine, Keimyung University, Daegu, KR; 4 Department of Surgery, Daegu Catholic University Medical Center, Catholic University of Daegu School of Medicine, Daegu, KR

**Keywords:** appendicitis, single-incision laparoscopic appendectomy, conventional laparoscopic appendectomy

## Abstract

**Introduction:**

Although single-incision laparoscopic appendectomy (SILA) was introduced decades ago, it is still considered a difficult technique to perform compared to conventional laparoscopic appendectomy (CLA). In addition, controversy about the benefits of SILA compared to CLA abound and no definite criteria for choosing SILA over CLA in patients with appendicitis currently exist. Therefore, we have planned a multi-center randomized controlled trial to compare SILA with CLA in terms of cosmetic satisfaction and pain reduction.

**Methods and analysis:**

Patients diagnosed with appendicitis at the participating centers will be recruited and allocated into either a CLA or an SILA groups using a 1:1 randomization. Patients in the CLA group will receive a conventional 3-port laparoscopic appendectomy and patients in the SILA group will receive a laparoscopic appendectomy using a single-incision at the umbilicus. The primary trial endpoint is cosmetic satisfaction assessed using the Patients and Observer Scar Assessment Scale (POSAS) administered 6 weeks post-surgery. Secondary trial endpoints include cosmetic satisfaction assessed via the Body Image Questionnaire, pain levels assessed via the Visual Analog Scale and International Pain Outcomes questionnaire, and the presence of postoperative complications. The target sample size of this superiority trial is 120 patients, as this will provide 80% power at the 2.5% level of significance to detect a 3-point difference in POSAS.

**Discussion:**

The results of this planned multi-center randomized controlled trial will provide substantive evidence to help surgeons choose when to use SILA over CLA in patients with appendicitis.

**Ethics and dissemination:**

This trial was approved by the institutional review board at Daegu joint on February 27, 2020 (No: 19-12-001-001) and registered with the clinical research information service (CRIS) (KCT0005048). The results of the study will be published and presented at appropriate conferences.

**Highlights:**

- To investigate the clinical benefits comparing between single incision laparoscopic appendectomy and conventional laparoscopic appendectomy
- To assess the pain and cosmetic satisfaction through quantitative scales, Patient-Reported Outcomes Measures (PROMs), International Pain Outcome (IPO) Questionnaire, the Patient and Observer Scar Assessment Scale (POSAS), and the Body Image Questionnaire (BIQ)
- To help surgeons choose when to use single incision laparoscopic appendectomy in patients with appendicitis.

## Background

Acute appendicitis is one of the most common causes of emergency gastrointestinal surgery worldwide. Even though controversy regarding the medical management of acute appendicitis using antibiotics exists, appendectomy is currently considered the gold standard treatment.

Open appendectomy rather than laparoscopic appendectomy was performed universally until the 1990s, even though Kurt Semm, a German gynecologist, first introduced laparoscopic appendectomy firstly in 1983 [1]. Currently, most appendectomies are laparoscopic because of the advantages including early recovery, less pain, and improved cosmetic satisfaction compared with open appendectomy [2].

A conventional laparoscopic appendectomy (CLA) usually requires insertion of three port trocars with two working ports and one camera port. Additionally, single-incision laparoscopic appendectomy (SILA) has become popular since it was first introduced in 1992 [3]. Recent meta-analysis reported that SILA is a safe and feasible procedure compare to CLA, though SILA is a considered more technically demanding than CLA [4,5,6].

Theoretically, SILA would be expected to produce less pain, encourage faster recovery, and result in better cosmetic satisfaction than CLA. However, several previous reports comparing SILA and CLA have yielded conflicting results and only a few studies have reported that SILA is superior to CLA with respect to pain and/or cosmesis [7,8,9,10,11]. One randomized control trial (RCT) failed to show the superiority of SILA over CLA with respect to pain and cosmesis [12]. Furthermore, a recent meta-analysis reported no differences in the pain and cosmesis scores between SILA and CLA [5,13]. Several studies, rather, have even reported that postoperative pain is more severe after SILA than after CLA [14,15].

Previous RCTs compared between SILA and CLA used simple visual analog score (VAS) for the assessment of both pain and cosmetic satisfaction. Results originated from this type of assessment have limitations with regard to their objectiveness.

There are several patient-reported outcomes measures (PROMs) used for assessing the pain and cosmetic satisfaction after surgery including the International Pain Outcome (IPO) Questionnaire [16], the Patient and Observer Scar Assessment Scale (POSAS) [17], and the Body Image Questionnaire (BIQ) [18]. However, there is a paucity of literature reporting outcomes comparing SILA and CLA using these tools.

Therefore, our trial aims to investigate the clinical benefits of SILA over CLA using these more objective PROMs for pain and cosmetic satisfaction.

## Methods

### Study design

**Figure 1:**
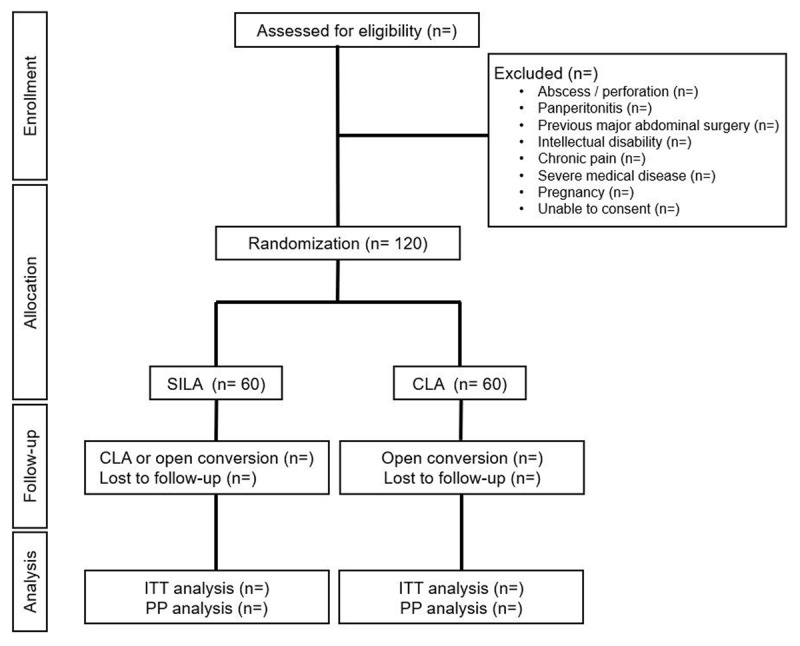
Study flow chart. CLA, Conventional Laparoscopic appendectomy; ITT, intention-to-treat; PP, per-protocol; SILA, Single-Incision Laparoscopic Appendectomy.

This study is a multi-center, prospective, open-label, randomized trial. Patients diagnosed with acute appendicitis at participating centers will be screened for study enrollment. Enrolled participants will be randomly allocated to either the SILA group or the CLA group. This study follows the recommendations of the SPIRIT (Standard Protocol Items: Recommendations for Interventional Trials) guidelines. The study flowchart is shown in ***Figure 1***.

### Inclusion criteria

Patients 19–75 years of age diagnosed with acute appendicitis who will receive a laparoscopic appendectomy will be recruited. Diagnosis of acute appendicitis is confirmed by abdominopelvic CT or ultrasonography performed on patients with clinically suspected appendicitis, and is defined by appendiceal diameter exceeding 6 mm with thickened and enhancing wall, and periappendiceal edema or fluid collection.

### Exclusion criteria

Patients who have one or more of the following will be excluded from this study: (1) patients with a suspected abscess or appendiceal perforation; (2) patients with symptoms of pan-peritonitis symptom; (3) patients with a history of major abdominal surgery; (4) patients who have an inability to express themselves due to conditions such as dementia or intellectual disability; (5) patients with chronic pain who need to take analgesics; (6) patients with severe medical disease such as pulmonary, cardiovascular, hepatic, or renal insufficiency; (7) Pregnancy; and (8) patients unable to provide consent.

### Interventions

#### SILA (Figure 2A)

A single 2cm incision will be made at the umbilical area and a custom multi-channel single port (Octoport, Dalim company, Co. Ltd., Korea) will be inserted into the incision site. There will be no restrictions placed on the type of laparoscopic instruments used, and all such decisions will be left to the discretion of the surgeon. The mesoappendix and appendiceal artery will be ligated and resected with an energy device or bipolar cauterization. The appendiceal base will be ligated with a loop tie or clip as per the surgeon’s preference. After the appendectomy, the facia will be closed with an absorbable suture and the skin will be closed using a nylon suture, an absorbable subcuticular suture, or a topical skin adhesive as per the surgeon’s preference.

**Figure 2:**
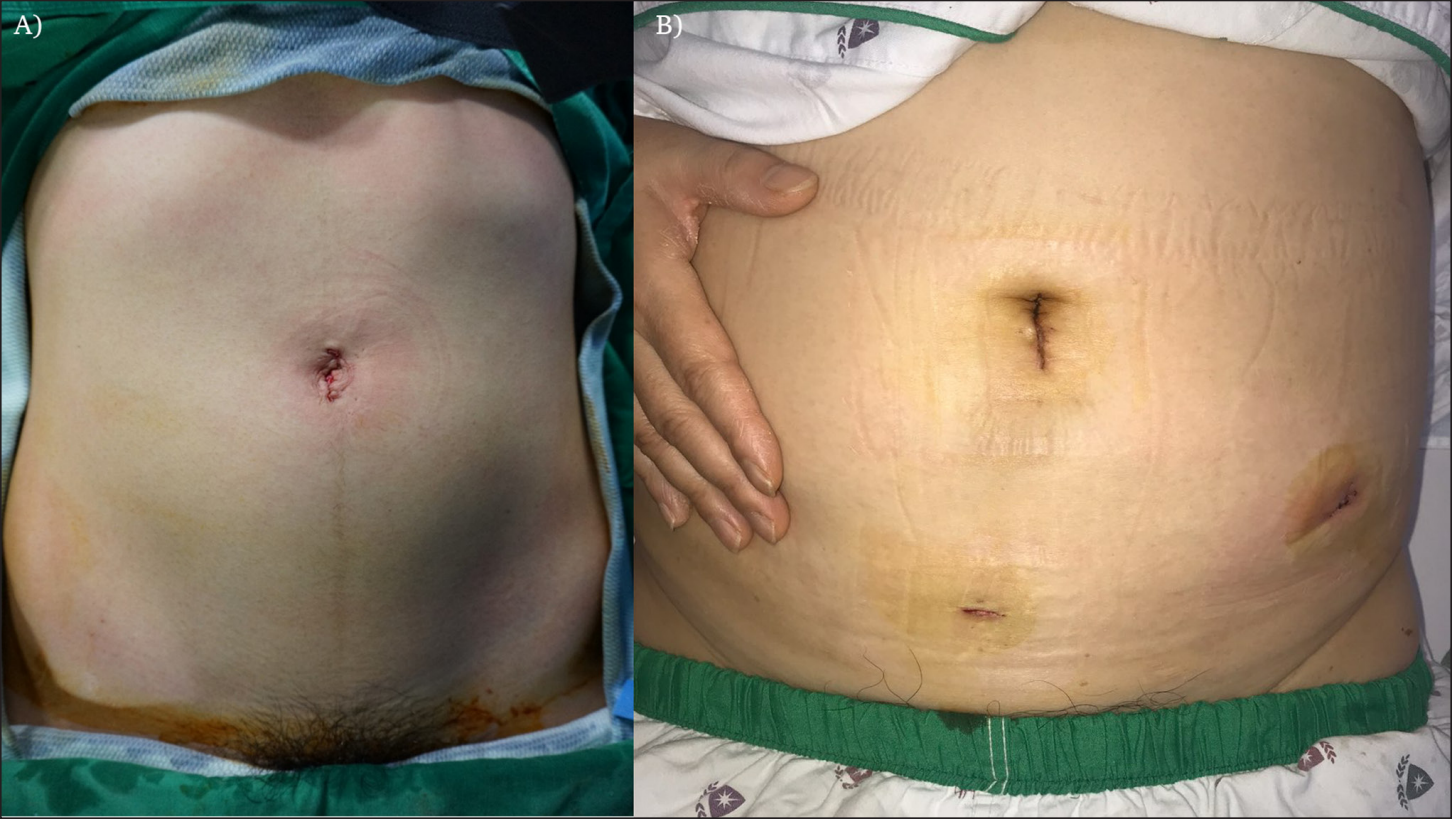
Incision and trocar position of SILA **(A)** and CLA **(B)**.

Additional port insertion or conversion to an open surgery will be possible at the surgeon’s discretion to ensure patient safety.

#### CLA (Figure 2B)

A standard three-trocar technique will be used with incisions made at the peri-umbilical, left lower quadrant, and supra-pubic sites. All other techniques for the appendectomy will be similar to those used in the SILA procedure.

### Randomization and sample size calculation

All participants will be randomized to either the SILA group or the CLA group in a 1:1 ratio. The randomization allocation will occur just to surgery using a computerized randomization system.

The target sample size will be 120 participants, as this will provide 80% power at the 2.5% (two-sided) level of significance to detect a three-point difference in the POSAS score between the SILA group and the CLA group at 6 weeks after surgery. Our target sample size allows for 10% attrition.

### Endpoints

The primary trial endpoint is cosmetic satisfaction at 6 weeks after surgery as measured by POSAS. The secondary endpoints are cosmetic satisfaction assessed via BIQ, pain assessed via the IPO questionnaire and VAS, and the presence of general postoperative complications.

### Data collection and management

**Figure 3:**
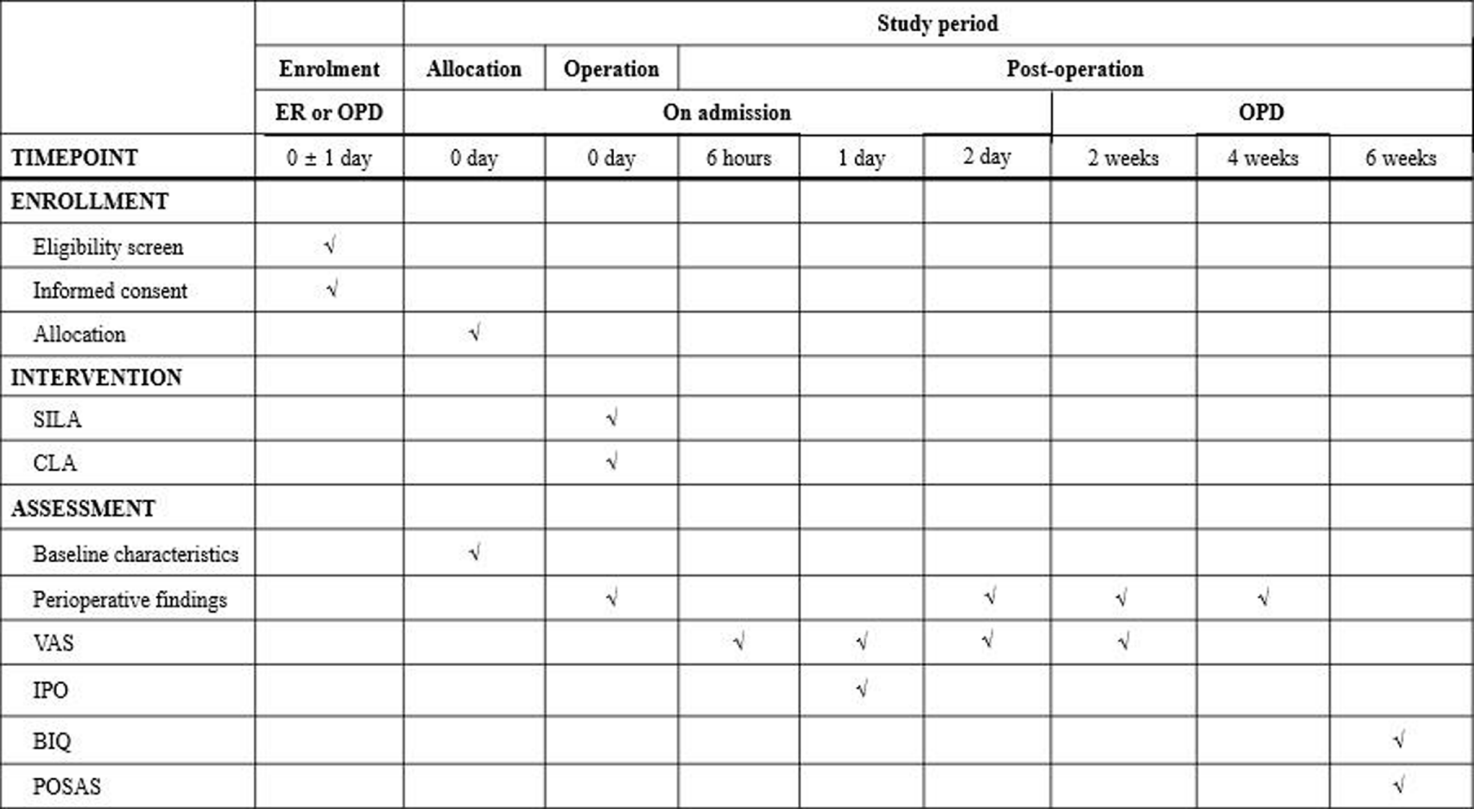
Schedule of assessment.

All data for this RCT will be collected after obtaining consent from the participants prior to surgery. All data will be recorded on a paper case report form as well as a digital record form. A participating surgeon or trained researching nurse will perform the postoperative interview to collect the necessary PROMs data (***Figure 3***).

#### Baseline characteristics

Baseline demographics for each participant such as age, sex, body mass index, medical history, etc. will be obtained prior to surgery but after informed consent is given.

#### Perioperative findings

Intraoperative findings including operative time, estimated blood loss, incision length, intraperitoneal findings, method of appendiceal base ligation, additional port insertion, conversion to open appendectomy, and skin suture technique will be recorded. Postoperative analgesics use, hospital stay, and morbidities within 30 days after surgery will be also collected.

#### PROMs for postoperative pain

VAS and IPO will be used for comparing postoperative pain between treatment groups. VAS will be obtained 6 hours after surgery, as well as on the morning of postoperative day 1 and 2. IPO will be obtained on postoperative day 1.

#### PROMs for cosmetic satisfaction

POSAS and BIQ will be obtained 6 weeks after surgery in the outpatient clinic. If any participant is unable to attend the clinic, a telephone interview will be permitted for completion of the survey.

#### Monitoring

All the data acquired for this study will be anonymized through the assignment of a trial identification number which will be used only for this study and accessed by only authorized persons. A study monitoring committee independent from the sponsor and investigator will check the study process and participants’ safety. Any adverse events related to the study will be reported to the study monitoring committee. All data will be stored for 3 years after completion of the study.

### Statistical analysis

All analyses of the primary and secondary endpoints will be conducted with the intention-to-treat population. A per-protocol analysis will also be performed for further comparisons. Normally distributed data will be conducted with Student’s t test. Non-normally distributed data will be examined using the Mann-Whitney U test. The chi-square or Fisher’s exact test will be used to examine categorical variables. All analyses will be performed using the SPSS statistical package version 21.0 (Chicago, IL, USA) and statistical significance will be declared for tests with p values < 0.05.

## Discussion

Laparoscopic abdominal surgery is generally regarded as a minimally invasive surgery with advantages such as less pain, faster recovery and better cosmetic results compared with open surgery. Many types of laparoscopic abdominal surgeries are currently used including single-incision procedure and conventional multi-port procedures [19,20,21]. Single-incision laparoscopic abdominal surgery is still a challenging procedure even among experienced laparoscopic surgeons [22,23]. Nevertheless, Appendectomy is one of abdominal surgery that is well-suited for a single-incision laparoscopic approach.

Surgeons and patients believe that reduced port surgery has advantages in terms of cosmetic satisfaction and pain reduction. This belief is based on the results of previous studies comparing open and laparoscopic surgery. In addition, among laparoscopic surgical techniques, SILA would be expected to outperform CLA with regard to cosmetic satisfaction and pain reduction because of further reductions in invasiveness. However, it has been difficult to prove the superiority of SILA over CLA because patient expectations are raised.

In this context, as mentioned above, there are controversies about the efficacy of SILA with respect to cosmetic satisfaction and pain reduction compare to CLA. However, previous studies including RCTs have used only a simple VAS and/or analgesic usage as indicators for pain reduction [8,10,24,25]. Although Anthony et al. [26] used a pain score that assessed overall pain as well as pain during specific activities (e.g. at rest, coughing for 10 time, after standing for 5 minutes), it was too detailed a score to get via PROM and provided only a subjective profile. Therefore, we decided not to use this pain evaluation scale.

For evaluation of cosmetic satisfaction, most previous studies used a subjective numeric rating from 5 to 100 [12,24,26,27]. The Vancouver Scar Scale and BIQ were used in two RCTs, but these studies were limited by their small sample size [8,10]. POSAS was used for the evaluation of cosmetic satisfaction in one recent study, but this study evaluated cholecystectomy and was not an RCT [28]. Therefore, we think that it is necessary to investigate the detailed differences in cosmetic satisfaction and pain reduction between SILA and CLA for appendectomy using objective scales in a study with an adequate sample size.

This study has some limitations even though it was designed as a prospective multi-center RCT. First, there are no Korean validated versions of the PROMs which will be used. However, investigators discussed the bases of clinical similarity and significance of the original version and translated the PROMs into Korean. In addition, all PROMS will be administered under the guidance of investigators or trained clinical nurses. Second, we will not be specifying which appendiceal base ligation or wound closer method must be used and instead will leave these decisions to the surgeon’s preference. This may act as a source of bias affecting the results. However, all the participating surgeons are colorectal surgery specialists who have performed hundreds of laparoscopic colorectal resections including SILA and CLA techniques. As a result, surgical skill should have a minimal impact on postoperative morbidities between surgeons.

In summary, this study is a prospective multi-center RCT designed to compare SILA and CLA with regard to cosmetic satisfaction and postoperative pain. We believe that the results of this study will clarify the efficacy of SILA for the treatment of acute appendicitis and will be helpful for determining which patients would benefit from SILA instead of CLA.

## Data accessibility statement

Data sharing is not applicable to this paper as no datasets were generated or analysed during the current study.

## Abbreviations

BIQ: Body Image Questionnaire; CLA: Conventional Laparoscopic Appendectomy; CRF: Case Report Form; CRIS: Clinical Research Information Service; IPO: International Pain Outcomes; POSAS: Patient and Observer Scar Assessment Scale; PROMs: Patient-Reported Outcomes Measures; RCT: Randomized controlled trial; SILA: Single Incision Laparoscopic Appendectomy; SPIRIT: Standard Protocol Items: Recommendations for Interventional Trials; VAS: Visual Analogue Scale.
